# Impaired skin microvascular endothelial reactivity in critically ill COVID-19 patients

**DOI:** 10.1186/s13613-022-01027-3

**Published:** 2022-06-13

**Authors:** Lisa Raia, Tomas Urbina, Paul Gabarre, Vincent Bonny, Geoffroy Hariri, Sebastien Ehrminger, Naïke Bigé, Jean-Luc Baudel, Bertrand Guidet, Eric Maury, Jeremie Joffre, Hafid Ait-Oufella

**Affiliations:** 1 Medical Intensive Care Unit, Saint Antoine University Hospital, APHP, Sorbonne University, 75012 Paris, France; 2 Pierre Louis Institute of Epidemiology and Public Health, Sorbonne University, Inserm U1136, Paris, France; 3 Centre de Recherche Saint-Antoine, Sorbonne University, Inserm U938, 75012 Paris, France; 4 Paris Cardiovascular Research Center, Paris University, Inserm U970, Paris, France

**Keywords:** SARS-CoV-2, Endothelial reactivity, Tissue perfusion, Intensive care medicine

## Abstract

**Background:**

Some clinical and histological studies have reported that SARS-CoV-2 infection may damage the endothelium. However, the impact of this virus on endothelial function in vivo remains poorly characterized. In this single-center pilot observational study, we performed iontophoresis of acetylcholine coupled with Laser doppler to investigate microvascular endothelial reactivity in COVID-19 patients compared to patients with non-COVID-19 bacterial pneumonia (NCBP) patients.

**Results:**

During three consecutive months, 32 COVID-19 patients and 11 control NCBP patients with acute respiratory failure were included. The median age was 59 [50–68] and 69 [57–75] years in COVID-19 and NCBP groups, respectively (*P* = 0.11). There was no significant difference in comorbidities or medications between the two groups, except for body mass index, which was higher in COVID-19 patients. NCBP patients had a higher SAPS II score compared to COVID-19 patients (*P* < 0.0001), but SOFA score was not different between groups (*P* = 0.51). Global hemodynamic and peripheral tissue perfusion parameters were not different between groups. COVID-19 patients had significantly lower skin microvascular basal blood flow than NCBP patients (*P* = 0.02). In addition, endothelium-dependent microvascular reactivity was threefold lower in COVID-19 patients than NCBP patients (*P* = 0.008).

**Conclusions:**

Both baseline skin microvascular blood flow and skin endothelial-dependent microvascular reactivity were impaired in critically ill COVID-19 patients compared to NCBP patients, despite a lower disease severity score supporting a specific pathogenic role of SARS-CoV-2 on the endothelium.

**Supplementary Information:**

The online version contains supplementary material available at 10.1186/s13613-022-01027-3.

## Introduction

The COVID-19 outbreak, caused by the Severe Acute Respiratory Syndrome coronavirus 2 (SARS-CoV-2), has affected unprecedently all regions of the world. About 5% of infected patients require intensive care unit (ICU) admission because of acute respiratory failure but also extra-respiratory disorders, including acute kidney injury or myocarditis [1, 2]. Frequent (arterial and) venous thrombosis affecting large vessels and microcirculation have been observed in COVID-19 patients suggesting that the SARS-CoV-2 virus may impact endothelial cell function and/or survival. Such a hypothesis was supported by 1/experimental studies showing that the SARS-CoV-2 bind to the angiotensin-converting enzyme 2 receptors, widely expressed on lung epithelial cells and vascular endothelial cells [3] 2/ histological analysis showing endothelium damage, named endothelitis, in several organs of COVID-19 non-survivors [4] 3/ sublingual videomicroscopy showing the accumulation of leukocytes and red blood cell microaggregates in the microcirculation of COVID-19 patients [5] despite normal red blood cells deformability [6]. However, the impact of SARS-CoV-2 infection on endothelial function in severely ill COVID-19 patients remained unknown.

This prospective pilot observational study compared skin microvascular endothelial reactivity in critically ill COVID-19 and patients admitted in the ICU for non-COVID-19 bacterial pneumonia (NCBP).

## Materials and methods

### Patients

We conducted a prospective, observational study in an 18-bed ICU in a tertiary teaching hospital in France between January and March 2021. We included consecutive COVID-19 adult patients during the first 24 h of ICU admission for acute respiratory failure. SARS-CoV-2 infection was confirmed by real-time reverse transcriptase–polymerase chain reaction (RT–PCR) assay on nasal or pharyngeal samples, without evidence of bacterial co-infection (sputum or BAL fluid culture negative at 48 h). COVID-19 patients with highly suspected or proven bacterial co-infections were excluded. Patients admitted to our ICU for severe bacterial pneumonia and tested negative for SARS-CoV-2, during the same period, were used as controls (NCBP group). In this pilot study, we exclusively focused on patients who did not require invasive mechanical ventilation to limit confounding factors. Additional exclusion criteria were: the need for vasopressor at the time of inclusion, skin lesions in the forearm recording area, and agitation.

### Assessment of endothelium-dependent skin microvascular vasoreactivity

The skin microvascular endothelial reactivity was assessed in the forearm area by acetylcholine (Ach) iontophoresis coupled with Laser doppler. This non-invasive technique allows transdermal diffusion of acetylcholine across the skin to subcutaneous capillaries. An electrical current is applied onto the skin, creating local differences in electrical potential and the active migration of ions and molecules bearing a net electrical charge through epithelial layers. At the endothelial level, acetylcholine promotes the production of nitric oxide (NO) by the endothelial NO-synthase, which diffuses to smooth muscle cells. This results in endothelium-dependent vasodilatation and increased blood flow.

The iontophoresis drug delivery chamber loaded with 80 uL of acetylcholine was attached to the volar side of the non-dominant forearm. The positive lead of the current source was attached to the drug delivery chamber, and the negative lead to a conductive hydrogel pad fixed 5 to 10 cm above.

A laser-Doppler flowmetry probe (Periflux 5000, Perimed) was used with a current-controlled delivery device (Perilont, Perimed). After recording the baseline blood flow for 60 s, three successive applications of acetylcholine were made, every minute, using anodal current (0.12 mA for 12 s each). Laser Doppler flowmeter signals were recorded continuously using an interface computer with acquisition software (Perisoft, Perimed). Skin blood flow was recorded during 10 min after the first iontophoresis of acetylcholine. Skin blood flow measurements were quantified as the baseline blood flow (expressed as Perfusion Units) and the maximal increase (peak value). The endothelial reactivity was quantified by the area under the curve (AUC) of the blood flow curve within a standardized 10-min recording [7–9].

Skin microvascular endothelial reactivity was performed within the 24 h following ICU-admission by an independent physician who did not participate in the patient’s care.

### Data collection

The following patients’ characteristics were recorded: age, gender, comorbidities and usual medication, the severity of illness evaluated by Simplified Acute Physiology Score II (SAPS II) and Sequential Organ Failure Assessment (SOFA) at inclusion, the onset of symptoms, respiratory support, use of vasopressor. We collected at inclusion global hemodynamic parameters (mean arterial pressure [MAP], heart rate [HR] and, cardiac index [CI]) and peripheral tissue perfusion parameters (Mottling score, skin temperature, Central-to-skin temperature gradient, arterial lactate level and urine output). Cardiac output and cardiac index were measured using transthoracic echocardiography (Vivid 7 Dimension’06, GE Healthcare). Biological parameters were also collected at inclusion. In this observational study, the Ach iontophoresis result did not imply any specific intervention or deviation from the standard of care procedures. The protocol was approved by the ethical committee of *Société de Réanimation de Langue Française (CE SRLF 21–59).*

### Statistical analysis

Patient characteristics were expressed as median (25th–75th interquartile ranges) or number and percentage as appropriate. Comparisons between groups were made by *chi-square* test for discrete variables and Mann–Whitney test for continuous variables. We used Spearman's *Rho* test to measure the strength of association between two variables. Statistical significance was defined as a two-sided *P* value of less than 0.05. All statistical analyses were performed with Prism, v7.0 (Graph Pad Software^®^, Inc., La Jolla, CA). No power calculation was performed in this explorative pilot study which was designed to include all consecutive patients over a 3 months period.

## Results

### Patients’ characteristics

During the 3-month inclusion period, 44 COVID-19 patients were admitted to our ICU for acute respiratory failure. Twelve patients were excluded because of proven bacterial co-infection. Finally, 32 COVID-19 patients and 11 patients with non-COVID-19 bacterial pneumonia (NCBP) were included. No significant difference was observed between the two groups regarding demographics, comorbidities or regular chronic medications (Table 1). The body mass index was lower in NCBP patients (*P* = 0.02). SAPS II was higher in NCBP patients when compared to COVID-19 patients (46 [32–51] vs. 23 [18–30], *P* < 0.01) but no significant difference for SOFA score (4 [3, 4] vs. 4 [2–5.3], *P* = 0.51) was observed. Regarding respiratory support at inclusion in the COVID-19 group, 25 (78.1%) patients were treated with high flow nasal cannula oxygen therapy (HFNC), associated with non-invasive ventilation in 16 (50%) patients, whereas only one patient received HFNC and none non-invasive ventilation in the NCBP group. Other patients received only oxygen through a non-rebreather mask or nasal cannula. The median Pao2/FiO2 ratio at inclusion was 239 [165–287] in the NCBP group vs. 128 [81–157] in the COVID-19 group (*P* < 0.01) (Table 1).

**Table 1:** Patients’ characteristics

Patients ‘characteristics n (%) or Median [IQR]	Non-COVID-19 bacterial pneumonia *N* = 11	COVID-19 *N* = 32	*P* value
Age, years	69 [57–75]	59 [50–68]	0.11
Men	7 (63.6)	21 (65.6)	1.00
BMI, kg/m^2^	22.5 [20.1–28.5]	26.6 [24.5–34.5]	**0.03**
SAPS II score	46 [32–51]	23 [18.3–30]	** < 0.001**
SOFA score	4 [2–5.3]	4 [3, 4]	0.51
Respiratory SOFA score	2 [2, 3]	3 [3, 4]	** < 0.001**
Sepsis (sepsis III criteria)	10 (90)	32 (100)	**0.94**
Comorbidities			
Active smokers	5 (45.5)	4 (12.5)	**0.03**
Hypertension	3 (27.3)	13 (40.6)	0.49
Diabetes mellitus	2 (18.2)	6 (18.8)	1.00
Vascular disease	1 (9.1)	3 (9.4)	1.00
Cancer/hemopathy	2 (18.2)	2 (6.3)	0.56
Time between, days			
First symptoms to ICU	2 [2–7]	8.5 [6–10]	** < 0.001**
First symptoms to inclusion	4 [2–7]	9 [7–10]	** < 0.001**
Respiratory support			
Nasal cannula/non-rebreather mask	10 (90.9)	7 (21.9)	** < 0.001**
CPAP or BiPAP	0 (0)	16 (50)	** < 0.001**
High flow nasal cannula	1 (9.1)	25 (78.1)	**0.003**
Biologicals			
Leukocyte count, G/L	8.63 [1.16–14.59]	7.745 [4.745–11.3]	0.90
Lymphocyte count, G/L	1.28 [1.07–1.88]	0.70 [0.54–0.89]	**0.01**
Platelet count, G/L	147[99–266]	197.5 [171–300.75]	0.07
Creatinine, µmol/L	71 [52–109]	62 [53–76]	0.22
Fibrinogen, g/L	7.09 [4.8–7.79]	6.76 [5.88–7.68]	0.67
C-reactive protein, mg/L	240.6 [95.6–302.3]	73.7 [42.55–121.2]	**0.03**
Procalcitonin, µg/L	7.68 [2.8–35.8]	0.15 [0.1–0.27]	** < 0.001**
Arterial blood gases			
pH	7.42 [7.42–7.44]	7.48 [7.45–7.51]	**0.002**
PaCO2, mmHg	37 [31–38]	34 [31–37]	0.22
Pa02/FiO2 ratio	239.5 [165.3–286.8]	128 [81–157]	**0.002**
Arterial lactate, mmol/L	1.6 [1, 2]	1.3 [1.1–1.7]	0.45

ACE, Angiotensin-converting enzyme; CPAP, Continuous Positive Airway Pressure; ICU, Intensive Care Unit; HIV, human immunodeficiency virus; SAPS, Simplified Acute Physiology Score; SOFA, Sequential Organ Failure Assessment

Biological parameters are depicted in Table 1. No significant difference was found between NCBP and COVID-19 patients for leucocytes count, C-reactive protein, or fibrinogen. Procalcitonin levels were significantly higher in NCBP patients (7.68 [2.8–35.8] vs. 0.15 [0.1–0.27], *P* < 0.01). Treatments received on the day of the iontophoresis are reported in Additional file 2: Table S1. Length of ICU stay was 5 [3–7] days in NCBP and 8 [5–14] days in COVID-19 group (*P* = *0.03*). In ICU mortality rate was 9% in NCBP group and 15.6% in COVID-19 group (*P* = *0.67*).

### Hemodynamics and tissue perfusion

At inclusion, no patient received vasopressor. There were no significant differences between the two groups regarding mean arterial blood pressure, heart rate, or cardiac index (Table 2). Arterial lactate level was similar between the groups (1.6 [1, 2] in NCBP patients vs. 1.3 [1.1–1.7] mmol/l in COVID-19 patients, *P* = *0.45*). The mottling score as well as the body-to-skin temperature gradient were not different between the two groups (Table 2).

**Table 2:** Global hemodynamic, tissue perfusion and iontophoresis parameters

Variables, *n* (%) or Median [IQR]	Non-COVID-19 bacterial pneumonia *N* = 11	COVID-19 *N* = 32	*P* value
Hemodynamic parameters			
Heart rate, bpm	97 [80–120]	84 [76–95]	0.15
Systolic blood pressure, mmHg	132 [120–145]	128 [115–136]	0.87
Diastolic blood pressure, mmHg	70 [62–94]	75 [68–81]	0.61
Mean arterial pressure, mmHg	89 [83–107]	89 [84–96]	0.66
Cardiac index, L/min/m^2^	2.8 [2.2–3.6]	2.9 [2.4–3.3]	0.90
Tissue perfusion parameters			
Core temperature, °C	37.5 [37–38.5]	37.1 [36.7–37.5]	0.09
Skin temperature, °C	31.4 [29.8–32.2]	31.4 [29.9–32.2]	0.79
Core-skin temperature gradient	6.3 [5.7–7.2]	5.7 [4.7–7.2]	0.39
Mottling score, *n* (%)			
0	8 (100)	23 (95.8)	1.00
1	0 (0)	1 (4.2)	
> 1	0 (0)	0 (0)	
Acetylcholine iontophoresis			
Baseline, perfusion units	10.4 [9.4–12.1]	7.9 [5.5–9.9]	**0.02**
Area under the curve	14,280 [5038–19743]	3911 [1725–6318]	**0.008**

### Skin microvascular blood flow

**Fig. 1:**
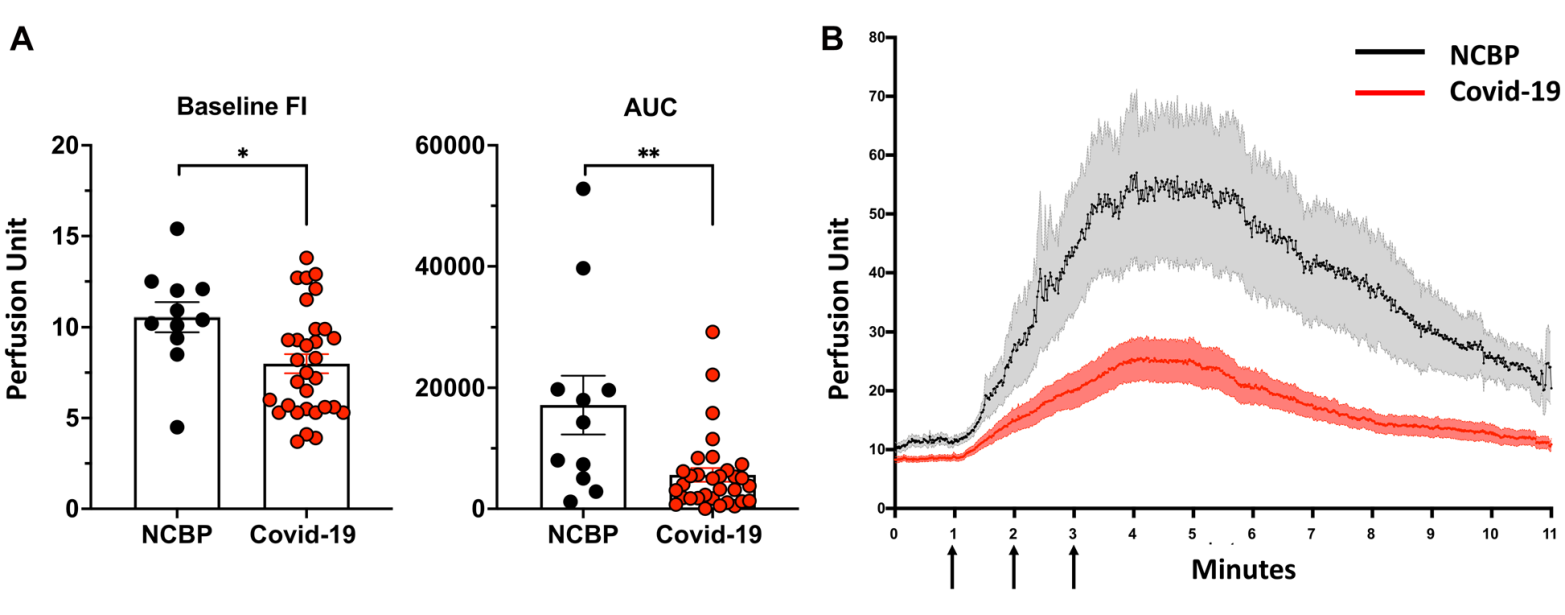
**A** Skin microvascular endothelial flow at baseline and reactivity defined by area under the curve in non-COVID-19 bacterial pneumonia (NCBP) and COVID-19 patients. **B** Skin microvascular blood flow in response to three stimulation of acetylcholine (arrows) in NCBP patients (grey) and COVID-19 patients (red), represented as mean + / SEM every second for 10 min after the first acetylcholine challenge

At baseline, the skin microvascular blood flow measured on the skin forearm area was significantly higher in NCBP compared to COVID-19 patients (10.4 [9.4–12.1] vs. 7.9 [5.5–9.9] PU, *P* = 0.02) (Table 2, Fig. 1A). After acetylcholine iontophoresis, skin microvascular blood flow significantly increases in both groups. However, the endothelium-dependent microvascular reactivity in NCBP patients was threefold higher compared to the COVID-19 group (AUC: 3911 [1725–6318] vs. 14,280 [5038–19743], (*P* < 0.01) (Table 2, Fig. 1A, B).

### Relationship between skin microvascular reactivity and duration of symptoms before admission

As expected, COVID-19 patients experienced longer symptomatic phase before ICU admission than NCBP patients (8.5 [6–10] vs. 2 [2–7] days, *P* < 0.01) (Table 1). Despite trend toward a negative association between impaired endothelial reactivity and time between first symptoms to ICU admission, this relationship did not reach statistical significance neither for COVID-19 (*P* = 0.06) nor NCBP patients (*P* = 0.63) (Additional file 1: Fig. S1A). Finally, when we pooled all included patients we found that the longer the time from first symptoms to ICU admission the lower the microvascular endothelial reactivity (R = -0.51, P = 0.002) (Additional file 1: Fig. S1B).

### Association of skin microvascular reactivity with biological, clinical parameters and outcome

Patients’ organ support therapy and outcome are reported in Additional file 3: Table S2. In COVID-19 patients, we did not observe any significant association between the SOFA score and skin microvascular vasodilation induced by Ach (*r* = 0.084, *P* = 0.65). Moreover, the skin endothelium-dependent microvascular reactivity was not associated with respiratory severity defined by the PaO2/FiO2 ratio (*r* = 0.25, *P* = 0.22) nor inflammatory biomarkers (Fibrinogen, D-dimers, CRP). Finally, we did not observe any significant relationship between Ach-induced microvascular reactivity and outcome neither in COVID-19 patients (AUC 3218 [622–17304] vs. 4040 [1726–6364], respectively, non-survivors (*N* = 5) and survivors (*N* = 27), *P* = 0.72) nor in NCBP patients (AUC 5038 vs. 16,133 [6223–24727], respectively, non-survivors (*N* = 1) and survivors (*N* = 10)).

## Discussion

Our study prospectively assessed skin endothelial-dependent microvascular reactivity in SARS-CoV-2 pneumonia and non-COVID-19 bacterial pneumonia. We first observed that the skin microvascular basal blood flow was significantly lower in COVID-19 patients compared to NCBP. Moreover, the endothelium-dependent microvascular reactivity was significantly lower in critically ill COVID-19 patients than NCBP despite higher disease severity.

As both a target organ and an effector, the endothelium has become of paramount interest in COVID-19 patients [10]. Here we explored the skin endothelium-dependent vasodilatation function in the forearm using iontophoresis of acetylcholine. We found altered microvascular reactivity in COVID-19 patients. This result is in line with previous experimental studies. Using either local thermal hyperemia [11] or iontophoresis of acetylcholine [12], moderate-to-severe patients with COVID-19 had an impaired endothelial reactivity. In these two studies, the COVID-19 population was heterogeneous with mechanically ventilated and non-mechanically ventilated patients, and compared to healthy volunteers. Similarly, Mesquida et al. reported impairment in microvascular reactivity using near infrared spectroscopy in COVID-19 patients compared to healthy controls, which was associated with acute respiratory distress syndrome (ARDS) severity [13]. In this study, the control group is constituted of septic patients with bacterial pneumonia non-mechanically ventilated without vasopressor or other organ failure. While it is well known that septic patients have endothelial dysfunction [14], we documented that COVID-19 patients have a more pronounced impaired endothelial function than NCBP patients despite lower severity. One remarkable finding of our study is that, although in the NCBP group microvascular reactivity was heterogeneous, all COVID-19 patients had a consistent impaired skin endothelium-dependent vasodilatation. In addition, we assessed the endothelial reactivity within the first 24 h following ICU admission, only in patients who did not require invasive ventilation. Despite that, we observed a substantial endothelial-mediated vasoreactivity impairment, suggesting an endothelial involvement in moderate to severe SARS-CoV-2 ARDS without secondary bacterial infection and prior to a possible multiorgan failure.

Using Ach iontophoresis, we indirectly explored the Nitric Oxide (NO) pathway as acetylcholine promotes endothelial production of NO. NO plays a crucial role in vascular homeostasis with its vasodilatory and anti-thrombotic actions [15]. In severe SARS-CoV-2 infection, besides the inflammatory and pro-oxidative environment, NO bioavailability is impaired [16]. In addition, SARS-CoV-2 infects host cells binding and downregulating of ACE-2, also reducing NO production [17]. These data suggest that NO insufficiency plays a substantial role in severe COVID-19. Indeed the damaged endothelium and reduced NO bioavailability contribute to inflammatory response and coagulopathy, thus amplifying the microvascular impairment in a vicious circle [18, 19]. Microvascular vasodilatation is ultimately carried out by the vascular smooth muscle cells (SMC). In this study, performing only Ach iontophoresis, we speculate that we indirectly explore the endothelial production of NO, but an impaired response could alternatively involve SMC dysfunction. Indeed, infected SMC [3] might become non-responsive to the EC-produced mediators in COVID-19 patients such as NO but also endothelium-derived hyperpolarizing factor or eicosanoids [20].

In our study, the basal skin blood flow was significantly lower in COVID-19 patients than in NCBP. This is in line with previous studies of sublingual microcirculation in which a reduced microvascular density in severe COVID-19 patients [21], in association with biomarkers of coagulopathy [22]. However, we did not find any significant differences between the COVID-19 group and the bacterial pneumonia group in terms of tissular perfusion parameters (mottling score, skin temperature). Of note, peripheral tissue perfusion was not severely impaired in included patients, probably because we did not include septic shock patients with vasopressors. Another possible explanation for this discrepancy is that microvascular blood flow and tissue perfusion parameters were not assessed in the same area, reflecting the tissue perfusion heterogeneity.

Microvascular alterations are strongly associated with mortality and organ failure in sepsis and septic shock [14, 23]. In our COVID-19 cohort, we did not observe a significant correlation between endothelial reactivity and organ failure (*i.e.* the coagulopathy [Fibrinogen, D-dimers] nor the severity of respiratory failure [PaO2/FiO2]). These results may be explained by a lack of power in a small cohort of non-invasive mechanically ventilated patients. In previous studies, endothelial dysfunction has been associated with disease severity and outcome [11]. For example, Rovas et al. reported that sublingual glycocalyx thickness, very difficult to assess in vivo, could predict the 60-day mortality [21]. Although these results need to be confirmed in larger clinical studies, we believe that microvascular endothelial dysfunction contributes to organ failure and death in severe COVID-19 and could be a potential target in further studies.

We acknowledge some limitations to this pilot translational observational study. First, this is a single-center study with a limited number of patients. Therefore, we could not identify an association between impaired endothelial reactivity and prognosis. In the same line, we did not find any significant relationship between time from first symptoms to ICU admission and endothelial reactivity in COVID-19 patients. In this observational study, we used a standardized and validated technique to explore the endothelium-dependent vasodilation in response to acetylcholine [24] performed for years in our ICU [14, 25]. However, we cannot infer the underlying mechanisms nor other endothelial functions impairment. We can speculate that endothelial dysfunction pre-existed in patients with COVID-19. Indeed, hypertension, diabetes and cardiovascular disease are common conditions predisposing to severe COVID-19 [26] and are known to be associated with endothelial dysfunction [27, 28]. Nevertheless, comorbidities were not significantly different between groups. Corticosteroids were more frequently used in COVID-19 patients which may be a potential confounder. Moreover, we chose to focus only on patients with an isolated respiratory failure and no need for invasive mechanical ventilation at inclusion. In multiorgan failure patients having support organ therapy, many factors could potentially impair the microvascular reactivity (vasopressors, acidosis, neuromuscular blockade [29], renal replacement therapy [30, 31] and make it difficult to attribute causation of endothelial dysfunction to the type of infection. In addition, using NCBP as a control group for COVID-19 is an arguable choice but severity comparison remains challenging because classical disease severity scores used in ICU such as SOFA or SAPS2 may not be necessarily appropriate in patients with SARS-CoV-2 infection. Acknowledging that there is no “ideal” control group for such explorative study and despite some significant differences in treatment at the time of admission, we assume that NCBP with isolated respiratory failure is conceptually appropriate. Finally, we measured skin microvascular endothelial reactivity at one single early timepoint and changes during ICU stay and more specifically time to recovery were not evaluated.

## Conclusions

In critically ill COVID-19 patients without invasive ventilation, we evidenced both a reduced baseline skin microvascular blood flow and a drastically impaired skin microvascular endothelium-dependent vasoreactivity compared to NCBP patients. These results support the hypothesis of a singular and clinically relevant SARS-CoV-2-associated endotheliopathy.

## Supplementary Information

**Additional file 1: Figure S1.** Correlation between the AUC of the microvascular blood flow following Acetylcholine iontophoresis and duration of symptoms before ICU admission in NCBP (Black, *P*=0.63) and COVID-19 (Red, *P*=0.061) (A) and in pooled NCBP/COVID-19 included patients (Black, *R*=-0.51, *P*=0.002)

**Additional file 2: Table S1.** Medication at admission

**Additional file 3: Table S2.** Organ support during ICU stay and outcomes

## Data Availability

The data sets used and/or analyzed during the current study are available from the corresponding author on reasonable request.

## Abbreviations

ACH: Acetylcholine
ARDS: Acute respiratory distress syndrome
AUC: Area under the curve
BMI: Body mass index
CI: Cardiac index
COVID-19: Coronavirus disease 2019
ECMO: Extracorporeal membrane oxygenation
HFNC: High flow nasal cannula oxygen therapy
HR: Heart rate
ICU: Intensive Care Unit
IQR: Interquartile range
MAP: Mean arterial pressure
NCBP: Non-COVID-19 bacterial pneumonia
NIRS: Near infrared spectroscopy
NO: Nitric oxide
RRT: Renal replacement therapy
SAPS II: Simplified Acute Physiology Score II
SARS-CoV-2: Severe acute respiratory syndrome coronavirus 2
SMC: Smooth muscular cells
SOFA: Sequential Organ Failure Assessment
