# Supplementary material for: Impaired skin microvascular endothelial reactivity in critically ill COVID-19 patients
Source: Ann Intensive Care. 2022 Jun 13;12:51. doi: 10.1186/s13613-022-01027-3 (PMC9188908; doi:10.1186/s13613-022-01027-3)
Supplement: Supplementary file 1 — Additional file 1: Figure S1. Correlation between the AUC of the microvascular blood flow following Acetylcholine iontophoresis and duration of symptoms before ICU admission in NCBP (Black, P=0.63) and COVID-19 (Red, P=0.061) (A) and in pooled NCBP/COVID-19 included patients (Black, R=-0.51, P=0.002) [file 13613_2022_1027_MOESM1_ESM.pptx]

## Slide 1
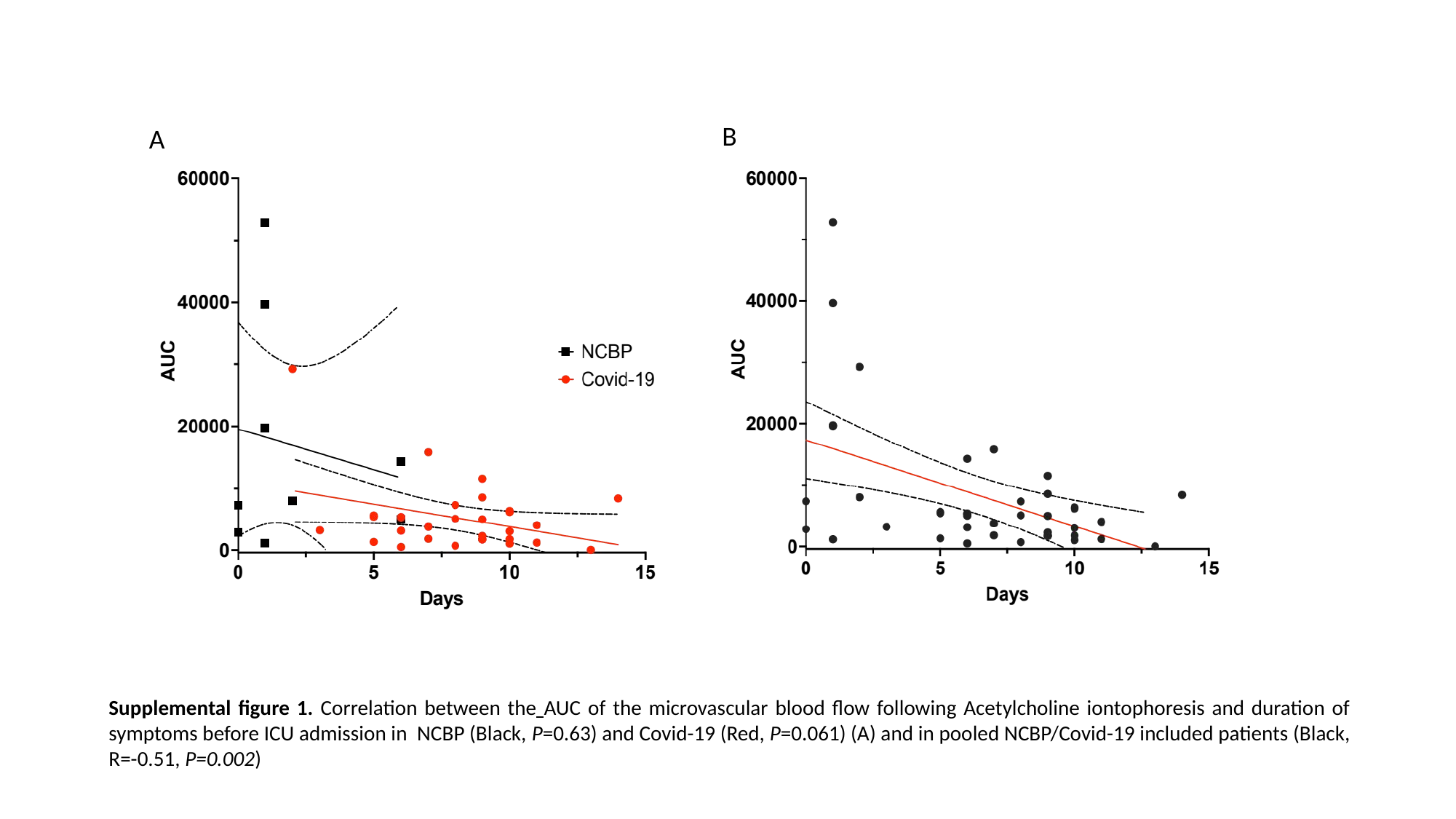

B
A
Supplemental figure 1. Correlation between the AUC of the microvascular blood flow following Acetylcholine iontophoresis and duration of symptoms before ICU admission in NCBP (Black, P=0.63) and Covid-19 (Red, P=0.061) (A) and in pooled NCBP/Covid-19 included patients (Black, R=-0.51, P=0.002)
