# Supplementary material for: Impaired skin microvascular endothelial reactivity in critically ill COVID-19 patients
Source: Ann Intensive Care. 2022 Jun 13;12:51. doi: 10.1186/s13613-022-01027-3 (PMC9188908; doi:10.1186/s13613-022-01027-3)
Supplement: Supplementary file 2 — Additional file 2: Table S1. Medication at admission [file 13613_2022_1027_MOESM2_ESM.docx]

| **Treatment, n(%)** | **NCBP**  **N=11** | **Covid-19**  **N=32** | ***P*** |
| --- | --- | --- | --- |
| **Therapeutic anticoagulation** | 2 (18.1) | 32 (100) | <0.0001 |
| **Dexamethasone/ Methylprednisolone** | 0 (0) | 32 (100) | <0.0001 |
| **Antibiotics** | 11 (100) | 2 (6.2) | <0.0001 |
| **Tocilizumab** | 0 (0) | 2 (6.2) | 1.00 |
| **Convalescent plasma** | 0 (0) | 1 (3.1) | 1.00 |
| **Statins** | 2 (18.2) | 7 (21.9) | 1.00 |
| **ACE-2 inhibitors** | 3 (27.3) | 9 (28.1) | 1.00 |
| **β-blockers** | 4 (36.4) | 8 (25) | 0.70 |

Additional file 2: Table S1: Medication at admission
