# Supplementary material for: Impaired skin microvascular endothelial reactivity in critically ill COVID-19 patients
Source: Ann Intensive Care. 2022 Jun 13;12:51. doi: 10.1186/s13613-022-01027-3 (PMC9188908; doi:10.1186/s13613-022-01027-3)
Supplement: Supplementary file 3 — Additional file 3: Table S2. Organ support during ICU stay and outcomes [file 13613_2022_1027_MOESM3_ESM.docx]

|  | **NCBP**  **N=11** | **Covid-19**  **N=32** | ***P*** |
| --- | --- | --- | --- |
| **Invasive MV, n (%)** | 3 (27) | 7 (22) | 0.70 |
| **Invasive MV duration (days, median [IQR])** | 3 [1-13] | 23 [11-38] | 0.08 |
| **Vasopressors, n (%)** | 4 (36.4) | 5 (15.6) | 0.20 |
| **Vasopressors duration**  **(days, Median [IQR])** | 0 [0-1] | 0 [0-1] | 0.06 |
| **RRT, n (%)** | 0 (0) | 0 (0) | - |
| **Lenght of stay (days, Median [IQR])** | 5 [3-7] | 8 [5-14] | **0.03** |
| **Death in ICU, n (%)** | 1 (9) | 5 (15.6) | 1.00 |

Additional file 3: Table S2: Organ support therapy during ICU stay and outcomes

Abbreviations: ICU: Intensive Care Unit; RRT: Renal Replacement Therapy; MV: Mechanical ventilation
